# Investigating the prostate specific antigen, body mass index and age relationship: is an age–BMI-adjusted PSA model clinically useful?

**DOI:** 10.1007/s10552-016-0827-1

**Published:** 2016-11-09

**Authors:** Sean Harrison, Kate Tilling, Emma L. Turner, J. Athene Lane, Andrew Simpkin, Michael Davis, Jenny Donovan, Freddie C. Hamdy, David E. Neal, Richard M. Martin

**Affiliations:** 1 School of Social and Community Medicine, University of Bristol, Canynge Hall, 39 Whatley Road, Bristol, BS8 2PS UK; 2 Nuffield Department of Surgery, John Radcliffe Hospital, University of Oxford, Oxford, OX3 9DU UK; 3 University Department of Oncology, Addenbrooke’s Hospital, Hills Road, Cambridge, CB2 0QQ UK

**Keywords:** Prostate cancer, PSA, BMI, Age, Prostate cancer screening, PSA–BMI equation

## Abstract

**Purpose:**

Previous studies indicate a possible inverse relationship between prostate-specific antigen (PSA) and body mass index (BMI), and a positive relationship between PSA and age. We investigated the associations between age, BMI, PSA, and screen-detected prostate cancer to determine whether an age–BMI-adjusted PSA model would be clinically useful for detecting prostate cancer.

**Methods:**

Cross-sectional analysis nested within the UK ProtecT trial of treatments for localized cancer. Of 18,238 men aged 50–69 years, 9,457 men without screen-detected prostate cancer (controls) and 1,836 men with prostate cancer (cases) met inclusion criteria: no history of prostate cancer or diabetes; PSA < 10 ng/ml; BMI between 15 and 50 kg/m^2^. Multivariable linear regression models were used to investigate the relationship between log-PSA, age, and BMI in all men, controlling for prostate cancer status.

**Results:**

In the 11,293 included men, the median PSA was 1.2 ng/ml (IQR: 0.7–2.6); mean age 61.7 years (SD 4.9); and mean BMI 26.8 kg/m^2^ (SD 3.7). There were a 5.1% decrease in PSA per 5 kg/m^2^ increase in BMI (95% CI 3.4–6.8) and a 13.6% increase in PSA per 5-year increase in age (95% CI 12.0–15.1). Interaction tests showed no evidence for different associations between age, BMI, and PSA in men above and below 3.0 ng/ml (all *p* for interaction >0.2). The age–BMI-adjusted PSA model performed as well as an age-adjusted model based on National Institute for Health and Care Excellence (NICE) guidelines at detecting prostate cancer.

**Conclusions:**

Age and BMI were associated with small changes in PSA. An age–BMI-adjusted PSA model is no more clinically useful for detecting prostate cancer than current NICE guidelines. Future studies looking at the effect of different variables on PSA, independent of their effect on prostate cancer, may improve the discrimination of PSA for prostate cancer.

## Background

Prostate cancer is the second most common cancer in men worldwide, with 1.1 million new cases diagnosed in 2012 [1]. Although prostate cancer deaths are considerably fewer in number than incident cancers (307,000 deaths worldwide in 2012 [1]), prostate cancer is the fifth leading cause of death from cancer in men. Developed countries tend to have a higher incidence of prostate cancer than others, in part due to increasing and widespread testing for serum prostate-specific antigen (PSA) [1].

There is considerable controversy over the effectiveness of PSA screening for prostate cancer [2–6]. One issue affecting the accuracy of the PSA test is that it is influenced by many variables other than the presence of cancer, for example diet [7], ethnicity [8], genetic variation [9, 10], certain drugs [11–14], and non-malignant disease [15–17]. Several studies report an inverse relationship between PSA and body mass index (BMI) [18–23], which may explain the observed inverse relationship between BMI and incident prostate cancer [24]. However, studies suggest the observed inverse relationship is a result of confounders and is eliminated in multivariable models (adjusted for age, current statin, aspirin and other NSAID use, diabetes, and benign prostatic hyperplasia) [25]. Most studies also report an increase in PSA with age [18].

The explanation for the positive relationship between age and PSA is well understood; with age, the prostate enlarges and contains more PSA-producing tissue. Older prostates tend to leak more PSA because the normal physiologic barriers breakdown, which allows PSA to escape into capillaries leading to a slight increase in serum PSA concentration [26]. Here, the degeneration of prostatic cells is independent of prostate cancer; although degeneration may increase cancer risk over time, the increase in PSA caused by the degeneration is therefore not directly caused by cancer.

The explanation for the inverse relationship between BMI and PSA levels is more uncertain; one suggestion is that obesity causes hemodilution due to an increased plasma volume [22, 27, 28]; another is that reduced androgen levels and increased estrogen in overweight men cause lower circulating PSA levels [23]. The reduction in PSA caused by an increased BMI may lead to men not receiving a biopsy when a smaller man would, which may help explain the observed paradoxical inverse relationship of BMI with prostate cancer detection, but the positive relationship between BMI and increased prostate cancer mortality [24]. However, it is difficult to establish whether obesity affects prostate cancer directly or whether its effect on PSA means obese men are diagnosed later with an associated worse prognosis.

In current UK practice, PSA value thresholds are used when screening for prostate cancer to indicate further investigation by prostate biopsy. National Institute for Health and Care Excellence (NICE) guidelines advise using age-specific cutoff PSA measurements: for men aged 50–59 years ≥3.0 ng/ml; 60–69 years ≥4.0 ng/ml; 70 years and older ≥5.0 ng/ml [29]. However, BMI is not taken into account when considering whether to send a man for biopsy.

Prostate cancer risk calculators (ERSPC risk calculator 6 [30], the Prostate Cancer Prevention Trial (PCPT) risk calculator [31], and PSA-AV developed by Patel et al. [32]) take age into account when considering prostate cancer risk and are available in the literature and on the Internet. The PCPT risk calculator can also include BMI category [33]; the BMI-adjusted PSA for a man is calculated by multiplying his PSA by the ratio of the geometric mean of PSA for BMI <25 to the geometric mean of PSA for his BMI category.

The majority of men undergoing PSA tests in the UK are likely to be overweight or obese [34]. If the inverse PSA–BMI relationship is considerable, having a PSA threshold which decreases with increasing BMI (adjusting the PSA for BMI) may improve the accuracy of the test for detecting prostate cancer. The aim of this study was to examine the relationship between PSA and BMI in a large population-based study of men undergoing PSA tests, to derive a model to adjust the observed PSA for the relationship between BMI, age, and PSA and investigate whether an age–BMI-adjusted model for PSA would be clinically useful for detecting prostate cancer.

## Materials and methods

We conducted a cross-sectional study nested within ProtecT (Prostate testing for cancer and Treatment), a population-based randomized controlled trial which compares treatments for clinically localized prostate cancer. Study details are published elsewhere [35]. In brief, 225,000 men aged 50–69 in 9 centers across the UK were invited for PSA testing. Of the 111,000 men who attended a PSA test, 10,000 had a PSA ≥3.0 ng/ml: Those with a PSA <20 ng/ml were invited for a 10-core transrectal ultrasound-guided biopsy, a repeat PSA test, and a digital rectal exam, while men with a PSA over 20 ng/ml were referred to usual care. Those with clinically localized PCa were invited into the ProtecT randomized treatment trial, comparing radical surgery, radical conformal radiotherapy and active monitoring [35].

We selected all 3,096 men diagnosed with prostate cancer and a random sample of 18,231 men without prostate cancer and with full information on covariates. The random sample was generated prior to this study by matching cases with 6 men without prostate cancer from within the same 5-year age band and GP practice. All potential matches were ordered by computer-generated random numbers, and the first 6 controls were chosen as a match. Men without prostate cancer were defined as having received a PSA test with no subsequent histological confirmation of prostate cancer, either because they were not indicated for biopsy (PSA < 3.0 ng/ml) or because a 10-core biopsy was negative (PSA ≥ 3.0 ng/ml). All men provided written informed consent prior to inclusion in the study. Trent Multicentre Research Ethics Committee (MREC) approved the ProtecT study (MREC/01/4/025) and the associated Prostate Mechanisms of Progression and Treatment (ProMPT) study which collected height data (MREC/01/4/061) as part of a diet, health and lifestyle questionnaire. All data were anonymized prior to analysis.

### Inclusion and exclusion criteria

For men without prostate cancer, the inclusion criteria for this analysis were: age between 50 and 69 years with no previous history of prostate cancer or diabetes, a BMI between 15 and 50 kg/m^2^, and PSA ≤ 10.0 ng/ml. Of the 18,231 men without prostate cancer, 9,457 (52%) satisfied the inclusion criteria. Most excluded men lacked data on height (*n* = 6,916, 38%) to compute BMI, as height data were collected as part of a separate questionnaire not filled in by all ProtecT participants and thus were likely missing at random.

Men with diabetes and men with no information on diabetes status (*n* = 1,572, 8.6%) were excluded, because diabetes influences PSA levels, prostate cancer risk and is associated with BMI [20, 36]. Men with a PSA above 10.0 ng/ml were excluded (*n* = 33, 0.2%), as high PSA levels can be associated with increased risk of false negatives at prostate biopsy [37]. A value of 10.0 ng/ml was chosen as the threshold as it compromises between excluding those most as risk of having a false negative at prostate biopsy and keeping as many men as possible in the analysis.

For men with prostate cancer, the inclusion criteria for this analysis were: age between 50 and 69 years, no previous history of diabetes, and a BMI between 15 and 50 kg/m^2^. Of the 3,096 men with prostate cancer, 1,830 (59%) satisfied the inclusion criteria; most excluded men lacked height data to compute BMI (*n* = 931, 30%), and 280 men with diabetes were excluded (9%).

For all men without height data (*n* = 7,847), the average age and weight were 61.1 years and 86.4 kg, slight differences to men with height data (*n* = 13,412), 62.0 years and 85.0 kg (both *p* < 0.01). Men with prostate cancer were more likely to have height data, 70 versus 62% of men without prostate cancer. The mean PSA for men without prostate cancer but with height data was 1.39 ng/ml, slightly more than for men without prostate cancer and height data, 1.28 ng/ml (*p* < 0.01). Conversely, the mean PSA for men with prostate cancer and height data was 9.47 ng/ml, slightly less than for men with prostate cancer but without height data, 10.78 ng/ml (*p* = 0.21).

For men without prostate cancer only, men with diabetes (*n* = 1,029) had an average age of 63.2 years, BMI of 29.9 kg/m^2^, and PSA of 1.23 ng/ml, whereas men without diabetes (*n* = 11,772) had a lower average age (61.8 years), BMI (27.2 kg/m^2^), and higher PSA (1.39 ng/ml). Men with missing diabetes status (*n* = 8,526) had an average age of 61.3 years, BMI of 27.6 kg/m^2^, and PSA of 1.30 ng/ml.

### Statistical analysis

The BMI of each man was calculated by dividing their weight (in kg) by their height squared (in meters squared). 94% of the men were weighed at clinic by a nurse, but 6% of men only had self-reported weights in stones and pounds as part of a diet, health and lifestyle questionnaire. Height was self-reported in feet and inches. Self-reported weight and height measurements were converted from imperial to metric units when calculating BMI.

The data from the 9,457 men without prostate cancer and 1830 men with prostate cancer were used to derive a model associating age and BMI with PSA. A multiplicative model for PSA was assumed, where the relationships between PSA and age and BMI were dependent on an initial level of PSA; a change in age or BMI leads to a proportional change in PSA.

A multiplicative model is intuitively more appropriate than an additive model, as a man with a high PSA would be expected to have a larger change in PSA than a man with a low PSA for the same change in age or BMI. Multivariable linear regression of the natural logarithm of PSA against age, BMI, and case–control status, separately and together in univariable and multivariable models, was used to estimate the coefficients for the model. Case–control status was included as a covariate to account for any associations between age and BMI with prostate cancer, which would otherwise bias the results.

The model was used to derive “adjusted” PSAs, removing the effects of age and BMI on PSA separately and together. The adjustment changes the man’s observed PSA by an amount depending on the man’s observed PSA and the difference between the man’s age or BMI and the mean age and BMI in this study. The larger the difference between the man’s age or BMI from the study mean, and the larger the observed PSA, the more the observed PSA is altered. The adjusted PSA can be interpreted as what the man’s PSA would have been, if they had been of average age and BMI.

The equation to adjust PSA for age and BMI is shown here:

$${\text{Age}}/{\text{BMI}}\; {\text{adjusted}}\; {\text{PSA}} = \frac{\text{PSA}}{{e^{{\left( {a\; \times \;{\text{age}}_{\text{coef}} + b\; \times \;{\text{BMI}}_{\text{coef}} } \right)}} }}$$

where age–BMI-adjusted PSA is PSA adjusted for age and BMI, PSA is a man’s observed prostate-specific antigen in ng/ml, *a* is the difference between the man’s age and the population mean age in years, *b* is the difference between the man’s BMI and the population mean BMI in kg/m^2^, age_coef_ is the coefficient of age from our linear regression model, BMI_coef_ is the coefficient of BMI from our linear regression model, and *e* is the exponential function. This model assumes that the relationship between PSA and BMI and age is the same for men with and without prostate cancer, in this population.

The age–BMI-adjusted PSA was used to determine whether the adjustment of PSA for BMI and age was clinically useful for detecting prostate cancer. Sensitivity and specificity estimates were calculated for the use of PSA to detect prostate cancer at biopsy (see Box 1). These used thresholds of 3.0 and 4.0 ng/ml (commonly used thresholds in clinical practice in the UK) for observed PSA values, age-adjusted PSA, BMI-adjusted PSA, age- and BMI-adjusted PSA, and were also compared to the sensitivity and specificity of the UK NICE guideline thresholds for PSA testing [29].

### Sensitivity and specificity

The sensitivity was calculated as the number of men with diagnosed prostate cancer who had a PSA above the threshold level divided by the total number of men with diagnosed prostate cancer (PSA-positive cases/total cases). The specificity was calculated as the number of men without diagnosed prostate cancer who had a PSA below the threshold level divided by the total number of men without prostate cancer (PSA-negative controls/total controls).

Men with a PSA below 3 ng/ml may have undiagnosed prostate cancer, as they were not biopsied. In the Prostate Cancer Prevention Trial (PCPT), 17% (*n* = 759) of men with a PSA below 3 ng/ml had prostate cancer on biopsy [31]. As the ages and BMIs of men in the PCPT were different from ProtecT, it is difficult to estimate the number of men in ProtecT who had undiagnosed prostate cancer.

Because some men will not have been biopsied but will have undiagnosed prostate cancer, the calculated sensitivities in this study will be higher than the true sensitivities. Additionally, even men who received a biopsy may also have had undiagnosed prostate cancer due to the sensitivity of 10-core biopsy; Haas demonstrated that a 12-core biopsy of cadavers showed a sensitivity for all prostate cancers of between 36 and 53%, depending on sampling location within the prostate [38]. The sensitivity of 12-core biopsy rises to 80 and 85% for “clinically significant” and large (≥0.5 cm^3^) cancers, respectively. The specificity of biopsy was 99%, indicating there should be few incorrect diagnoses of prostate cancer.

Therefore, the total number of men with prostate cancer is underestimated in this study, both by men not receiving a biopsy and by the biopsy not detecting all cancers, so the sensitivity of each PSA test will be overestimated (see Box 2). The total number of men without prostate cancer is overestimated in this study by the same amount, but this is unlikely to affect the specificity as there are far more men without than with prostate cancer. However, the main analysis will focus on the 4.0 ng/ml threshold; as the biopsy threshold in ProtecT was 3 ng/ml almost all men who might change over a 4.0 ng/ml threshold will have been biopsied. Assuming there is no strong relationship between PSA and missing prostate cancer at biopsy, this means any change in sensitivity or specificity seen in the PSA models is unlikely to be affected by men with undiagnosed prostate cancer. Therefore, all PSA models in this study can be directly compared, even though they do not represent the true sensitivity of PSA as a test for prostate cancer.

NICE guidelines use different thresholds for different age groups, making it difficult to directly compare the sensitivity and specificity to the other models. To show clinical utility, our PSA models would require both a higher sensitivity and specificity; otherwise, there would be a trade of specificity for sensitivity, or vice versa. Therefore, the sensitivity of all models was compared at the same specificity seen when using the NICE guidelines; any model with a higher sensitivity would necessarily be more clinically useful. McNemar’s test was used to determine whether any model was preferred over the NICE guidelines [39] when the specificities of all models were equal.

As the sensitivities and specificities of the models are likely to be inaccurate, ROC curves (receiver operating characteristics) [40] and area under the curves (AUCs) were not generated.

Tenfold cross-validation [41] was used to determine whether the sensitivities and specificities of the adjusted PSAs were consistently better or worse than the NICE guideline thresholds for PSA testing. In tenfold validation, the dataset is split into 10 equal parts and each part of the dataset is considered the “validation” dataset, with the other 9 parts used to calculate the model that will be validated. This is repeated 10 times, until each part of the dataset has acted as the “validation” dataset. The sensitivities and specificities of each model were averaged across the 10 validations, with the mean and standard deviation recorded. These were then compared across models to give a robust indication of the performance of each model. The advantage of using tenfold cross-validation as opposed to split-cohort validation is that the training data can be as large as possible without compromising the robustness of the model performance in the testing data.

An interaction test [42] was performed to determine whether there was a difference in the associations between age, BMI, and PSA for men with PSA values above and below 3 ng/ml; this was to test whether the undiagnosed prostate cancers from men not being biopsied were causing any bias in the results. Multivariable regressions were performed as above, restricted to men with a PSA above and below 3.0 ng/ml separately. For both age and BMI, the difference in coefficients was divided by the combined standard error to give a *Z*-score, which was converted to a *p* value.

Gleason score was used as an additional outcome in men with diagnosed prostate cancer, and ordered logistic regression was used to determine whether age and BMI were associated with Gleason score in this population. In order to examine the sensitivity of conclusions to the relative proportions of cases and controls, we re-ran all primary analyses using only controls to derive the age- and BMI-adjusted PSA, and then examined the sensitivity and specificity of this model. A further sensitivity analysis used multiple imputations by the MICE system of chained equations to estimate missing BMI and diabetes data from weight, height, age, case–control status, diabetes status, and log-PSA to determine whether missing height/diabetes would likely have caused bias.

All analyses were performed using Stata 13.1 (StataCorp, TX).

**Box 1:** Definitions of sensitivity and specificity

Sensitivity—the true positive rate; the number of people ***with*** prostate cancer who had a PSA ***above*** the threshold level divided by the total number of people ***with*** diagnosed prostate cancer
Specificity—the true negative rate; the number of people ***without*** prostate cancer who had a PSA ***below*** the threshold level divided by the total number of people ***without*** prostate cancer

**Box 2:** Effect of undiagnosed prostate cancer on sensitivity and specificity of PSA testing for prostate cancer. Bold letters are the true number of men in each cell, and italic letters are the study number of men in each cell

	Prostate cancer	No prostate cancer
PSA ≥ 3.0 ng/ml	**A** *A* − *x*	**B** *B* + *x*
PSA < 3.0 ng/ml	**C** *0*	**D** *D* + *C*
Number of men with (left) and without (right) prostate cancer	**A** **+** **C** *A* − *x*	**B** **+** **D** *B* + *x* + *D* + *C*
**A** and **C** are the number of men truly with prostate cancer, **B** and **D** are the number of men truly without prostate cancer, and *x* is the number of men with prostate cancer but the biopsy missed the cancer. Men with a PSA above 3.0 ng/ml were biopsied, so any underestimation in **A** and overestimation in **B** is from biopsies that miss the cancer [38]. If there are ***x*** men with missed cancers, then **A** becomes **A** − ***x*** and **B** becomes **B** **+** ***x***. Men were not biopsied if their PSA was less than 3.0 ng/ml, so **C** becomes 0. Some men will have prostate cancer and a PSA less than 3.0 ng/ml [31], so **C** is underestimated and **D** becomes **D** **+** **C**. As **C** is 0, all calculated sensitivities [**A/(A** **+** **C)**] with a PSA threshold of 3.0 ng/ml or less will be 1. In this study, the sensitivity will always be overestimated, as the total number of men with prostate cancer (**A** **+** **C)** will always be underestimated. The specificity [**D/(B** **+** **D)**] may be over- or underestimated as both **B** and **D** are overestimated by different amounts, but overall the calculated specificity should not differ too much from the true specificity. Example: 2,500 men truly had prostate cancer (**A** **+** **C**), but 100 were missed at biopsy (***x***) and 500 had a PSA less than 3.0 ng/ml (**C**) so were not biopsied, and 7,500 men truly did not have prostate cancer (**B** **+** **D**); 1,000 of these men had a PSA greater than 3.0 ng/ml (**B**). The calculated sensitivity at 3.0 ng/ml would be (**A** ***x***)/(**A** − ***x***) = (2,000−100)/(2,000−100) = 1, but the true sensitivity would be **A**/(**A** **+** **C**) = 2000/2500 = 0.8; the sensitivity is overestimated. The calculated specificity at 3.0 ng/ml would be (**D** **+** **C**)/(**B** **+** ***x*** + **D** **+** **C**) = (6,500 + 500)/(1,000 + 100 + 6,500 + 500) = 0.86, and the true specificity would be **D/**(**B** **+** **D**) = 6,500/7,500 = 0.87; the specificity is very close to the true value.		

## Results

Summary demographics are presented in Table 1. The mean age for all men (*n* = 11,293), cases (*n* = 9,457), and controls (*n* = 1,836) was 61.7 (SD 4.9), 61.6 (SD 4.9), and 61.8 (SD 4.9) years, respectively. The mean BMI was 27.2 (SD 3.7), 27.2 (SD 3.8), and 27.1 (SD 3.6) kg/m^2^, respectively (*p* = 0.21). The median PSA was 5.0 ng/ml (IQR 3.7–8.0) in cases and 1.0 ng/ml (IQR 0.6–1.7) in controls (*p* < 0.0001). Ordered logistic regression of 1,830 men with a Gleason score with age and BMI showed both were weakly associated with Gleason score: age: coef = 0.03, *p* = 0.002; BMI: coef = 0.03, *p* = 0.04.

**Table 1:** Summary demographics of included participants; cases have a diagnosis of prostate cancer

	All	Cases	Controls	*p* value for difference
*n*	11,293	1,836	9,457	NA
Age (SD)	61.7 (4.94)	61.8 (4.90)	61.6 (4.95)	0.29
BMI (SD)	27.2 (3.73)	27.1 (3.57)	27.2 (3.76)	0.21
PSA (IQR)	1.2 (0.7–2.6)	5 (3.7–8.0)	1 (0.6–1.7)	<0.0001
BMI categories in kg/m^2^ [*n* (%)]				
<25	3,274 (29)	522 (28.4)	2,752 (29.1)	*p* for trend = 0.65
25–29.9	5,805 (51.4)	979 (53.3)	4,826 (51)	
>30	2,214 (19.6)	335 (18.3)	1,879 (19.9)	

In univariable models (*n* = 11,293), where log-PSA was regressed separately against age and BMI (with case–control status as a covariate), PSA increased by 13.55% (95% CI 12.01–15.11) per 5-year increase in age and decreased by 5.58% (95% CI 3.83–7.29) per 5 kg/m^2^ increase in BMI. When case–control status was omitted from the regression, PSA increased by 14.30% (95% CI 12.22–16.41) per 5-year increase in age and decreased by 6.55% (95% CI 4.24–8.81) per 5 kg/m^2^ increase in BMI.

In the multivariable model (*n* = 11,293), where log-PSA was regressed against BMI, adjusting for age and case–control status together, there was a 5.14% decrease in PSA per 5 kg/m^2^ increase in BMI (95% CI 3.41–6.84, *p* < 0.001) (Table 2). The BMI-adjusted results for age are not presented, as there is no plausible mechanism by which BMI can confound the age–PSA association.

**Table 2:** Results of linear regression of age and BMI against PSA separately (univariable) and together (multivariable)

Variable	*n*	Change in PSA (%)	Log(PSA) change per 5 unit increase in covariate		
			Coefficient	*p* value	95% CI
Univariable					
Age	11,293	13.55	0.127	<0.00001	0.113 to 0.141
BMI	11,293	−5.58	−0.057	<0.00001	−0.076 to −0.039
Multivariable					
Age	11,293	13.43	0.126	<0.00001	0.112 to 0.140
BMI	11,293	−5.14	−0.053	<0.00001	−0.071 to −0.035

The beta coefficients for change in log-PSA are equivalent to the logarithm of the multiplicative change in PSA per 5 unit increase in age–BMI, which has been expressed as a percentage change in the table. Small change in log-PSA is broadly interpretable as the percentage changes in PSA

*PSA* prostate-specific antigen, BMI body mass index, *SE* standard error

The sensitivities and specificities (as defined by the proportion of cases above the threshold PSA and the proportion of controls below the threshold PSA, respectively), of the different PSA models at 3.0 and 4.0 ng/ml thresholds, and the NICE guidelines (threshold dependent on age) are presented in Table 3. All sensitivities and specificities were calculated assuming no undiagnosed prostate cancers among men in the control group or misdiagnosis by biopsy and are thus inaccurate (the sensitivities are overestimates, and the specificities may be over- or underestimates, as explained in Box 2).

**Table 3:** Sensitivities (proportion of men diagnosed with prostate cancer (cases) above the threshold PSA) and specificities (proportion of men not diagnosed with prostate cancer (controls) below the threshold PSA) for prostate cancer detection at biopsy for different models

Model	Threshold 3.0 ng/ml		Threshold 4.0 ng/ml	
	Proportion of cases above threshold	Proportion of controls below threshold	Proportion of cases above threshold	Proportion of controls below threshold
Observed	1	0.931	0.687	0.964
Age–PSA	0.947	0.932	0.699	0.968
BMI–PSA	0.978	0.932	0.682	0.965
Age–BMI–PSA	0.942	0.932	0.708	0.967
NICE guidelines^a^	–	–	0.797	0.958

*PSA* prostate-specific antigen, *BMI* body mass index

^a^NICE guidelines–ages 50–59: 3.0 ng/ml, ages 60–69: 4.0 ng/ml

Compared to PSA alone, sensitivity was improved when adjusting PSA for BMI [by about 0.01 (1%)], but specificity worsened at both 3.0 and 4.0 ng/ml thresholds [by 0.003 (0.3%) and 0.001 (0.1%), respectively]. In comparison, adjusting PSA for age improved both the sensitivity and specificity at 4.0 ng/ml [by 0.026 (2.6%) and 0.002 (0.2%), respectively], but worsened both sensitivity and specificity at 3.0 ng/ml [by 0.021 (2.1%) and 0.003 (0.3%), respectively]. Adjusting PSA for both age and BMI showed similar results to adjusting solely for age, with the exception of improved sensitivity at 4.0 ng/ml, implying that adjusting for BMI as well as age did not materially improve the discrimination of PSA for prostate cancer.

When the specificity of the model PSAs was set to the same as the NICE guidelines (0.958), the sensitivities were all below that of the NICE guidelines: NICE: 0.797; PSA alone: 0.768; age-adjusted PSA: 0.794; BMI-adjusted PSA: 0.758; and age–BMI-adjusted PSA: 0.796. However, when each model was compared with the NICE guidelines using McNemar’s test, there was no evidence to say any model was better or worse at detecting prostate cancer: PSA alone *p* value 0.65; age-adjusted PSA *p* value: 1; BMI-adjusted PSA *p* value: 0.53; age–BMI-adjusted PSA *p* value: 1.

The tenfold cross-validation showed that the averaged sensitivities and specificities were very close to the sensitivities and specificities of the results from the main analysis. The standard deviations were low, indicating the models performed robustly across the ten validation sets (Table 4). Therefore, it is unlikely that the results from the main analysis are the product of validating the models in the same dataset used to develop the models.

**Table 4:** Averaged sensitivities and specificities for prostate cancer detection at biopsy for different models from tenfold cross-validation

Model	Threshold 3.0 ng/ml		Threshold 4.0 ng/ml	
	Proportion of cases above threshold (SD)	Proportion of controls below threshold (SD)	Proportion of cases above threshold (SD)	Proportion of controls below threshold (SD)
PSA	1 (0)	0.931 (0.011)	0.687 (0.022)	0.964 (0.005)
Age–PSA	0.946 (0.019)	0.933 (0.008)	0.699 (0.028)	0.968 (0.005)
BMI–PSA	0.978 (0.008)	0.932 (0.010)	0.683 (0.020)	0.965 (0.005)
Age–BMI–PSA	0.977 (0.008)	0.932 (0.010)	0.706 (0.028)	0.967 (0.005)
NICE guidelines^a^	–	–	0.798 (0.017)	0.958 (0.006)

*PSA* prostate-specific antigen, *BMI* body mass index

^a^NICE guidelines–ages 50–59: 3.0 ng/ml, ages 60–69: 4.0 ng/ml

The interaction test examining whether men with a PSA at or above 3.0 ng/ml (*n* = 2,489) had different estimates of the associations between age, BMI, and PSA to men with a PSA less than 3.0 ng/ml (*n* = 8,804) showed no evidence of an effect of not receiving a biopsy; the *p* value for the interactions with age and BMI, respectively, was 0.22 and 0.24.

In the sensitivity analysis using only men without prostate cancer (*n* = 9,457), multivariable linear regression showed a 5.51% decrease in PSA per 5 kg/m^2^ increase in BMI (95% CI 3.62–7.36) and univariable regression showed a 14.25% increase in PSA per 5-year increase in age (95% CI 12.54–15.98). Sensitivity and specificity for a threshold of 3 ng/ml were 0.900 and 0.898 and for a threshold of 4 ng/ml were 0.673 and 0.930, respectively. These results indicate that while the sensitivities and specificities were slightly less than the full model (3 ng/ml: 0.942, 0.932; 4 ng/ml: 0.708, 0.967), the association between PSA and age and BMI in men without prostate cancer in this population is similar to that in the entire population.

In the sensitivity analysis where BMI and diabetes data were imputed (*n* = 19,524), multivariable linear regression showed a 5.22% decrease in PSA per 5 kg/m^2^ increase in BMI (95% CI 3.67–6.80) and univariable regression showed a 13.33% increase in PSA per 5-year increase in age (95% CI 12.14–14.54). Averaged sensitivity and specificity over ten imputations for a threshold of 3 ng/ml were 0.941 and 0.938 and for a threshold of 4 ng/ml were 0.701 and 0.970, respectively. These values are very similar to the main analysis indicating missing height and diabetes data were unlikely to have biased the result.

## Discussion

This study has shown that in men aged 50–69 in the UK there is an inverse relationship between PSA and BMI, and a positive relationship between age and PSA. The magnitude and direction of effect of these relationships is consistent with previous research [15, 18–23]. In previous research, however, the relationship between BMI and PSA may have been assumed to be additive; this study considered the relationship to likely be multiplicative and has presented the results as such. This interpretation fits better with the proposed theories of why BMI may be associated with PSA.

Overall, adjusting for BMI did not materially improve the discrimination of PSA for detecting prostate cancer, as there was no evidence the NICE guidelines for screening for prostate cancer using age-band-specific PSA thresholds performed worse than our age-, BMI-, and age–BMI-adjusted PSA models. It is unlikely that the associations seen between age, BMI, and PSA are solely the result of associations with prostate cancer, as the likelihood tests showed no evidence that the results from men with a PSA less than 3.0 ng/ml (not biopsied) were different from men with a PSA more than 3.0 ng/ml (biopsied).

It is not clear from this study whether there is a causal relationship between BMI and PSA—the relationships between BMI, PSA, and prostate cancer are complex, and as not all men were biopsied it is impossible to disentangle relationships with underlying prostate cancer. Even if all men were biopsied, there would still be a risk of false negatives from missing the cancer on the biopsy [37, 38]. This, combined with the use of PSA testing as a means of screening for prostate cancer in general practice (as well as any variables which affect the overall risk of receiving a PSA test), makes it difficult for any study to examine the relationships surrounding PSA and prostate cancer risk.

The limitations of this study are recognized. The study population is large and taken from multiple centers across the UK, but will not necessarily be demographically diverse or applicable to other populations as almost all men classified themselves as “white” ethnicity. Although the tenfold cross-validation show very similar results to the main analysis, these results were not replicated in an external dataset. However, as internal validation usually shows some measure of overfitting, the conclusion that the NICE guidelines show better discrimination for prostate cancer should be robust.

PSA values were only taken on one day, but PSA levels are influenced by factors such as biological [43] or laboratory variation [44], inflammation [15] or infection [45]. Some evidence exists for seasonal variation in PSA [46]; however, a study using ProtecT data showed no variation in PSA due to time of year or amount of sunlight per day [47] and thus time of year should not have affected these results. As not all men were biopsied, the calculated specificities and sensitivities are likely overestimates as there were likely to be men with undiagnosed prostate cancer (Box 2). ROC curves and AUCs could not be generated as a result, and these results should not be compared with the true sensitivity and specificity of PSA as a test for detecting prostate cancer.

Height data were self-reported; this may have slightly biased the results if taller or shorter men were more likely to misreport their height. Additionally, a large number of men did not have a recorded height as they did not participate in the additional study that recorded height data; data were likely to be missing at random (i.e., it is unlikely that taller men were more likely to have missing height data). Multiple imputations showed that missing height data were unlikely to have biased the analysis as the models were very similar.

Men with diabetes were not included in the model, as BMI is a recognized risk factor for diabetes, and diabetes is associated with a lower PSA [48] and prostate cancer [36], so men with diabetes may obscure the relationship between BMI and PSA. Although there is conflicting evidence, smoking [49], exercise [50], and a low-fat diet [51] have all been associated with decreased PSA and BMI, and high alcohol intake and benign prostatic hypertrophy [52] have been associated with increased PSA and BMI [53]. These variables were not considered in this study, but these associations would indicate a positive relationship between BMI and PSA which is not observed in this study; thus, it is unlikely that the observed BMI–PSA relationship was biased away from the null by any of these variables.

## Conclusions

This study has described the relationship between age and BMI and PSA in men without diabetes. These relationships were used to adjust PSA to examine the potential clinical utility of an age–BMI-adjusted PSA, but this did not perform better than current NICE guidelines. More studies examining the effects of variables on PSA, independent of the effect on prostate cancer, could help to improve PSA testing for prostate cancer.
